# Role of the Wearable Defibrillator in Newly Diagnosed Heart Failure

**DOI:** 10.1007/s11897-018-0415-7

**Published:** 2018-10-23

**Authors:** David Duncker, Christian Veltmann

**Affiliations:** Rhythmology and Electrophysiology, Department of Cardiology and Angiology, Hannover Medical School, Carl-Neuberg-Str. 1, 30625 Hannover, Germany

**Keywords:** Wearable defibrillator, Newly diagnosed cardiomyopathy, Heart failure, Sudden cardiac death

## Abstract

**Purpose of Review:**

The wearable defibrillator (WCD) was shown to be safe and effective in detecting and terminating ventricular tachyarrhythmias and therefore allows temporary protection from sudden cardiac death. This review gives an overview of the current data on WCD in newly diagnosed cardiomyopathy.

**Recent Findings:**

Patients with newly diagnosed heart failure and reduced LVEF appear to have an increased risk of ventricular tachyarrhythmias, which may decrease over time when heart failure medication is optimized and left ventricular function improves. This was shown to apply for patients with ischemic and non-ischemic cardiomyopathy, including peripartum cardiomyopathy. Prolongation of the WCD period may support to further optimization of heart failure medication, by protecting the patient from sudden cardiac death during this time and to avoid untimely ICD implantation.

**Summary:**

The WCD should be considered in structured patient management for newly diagnosed heart failure during the early phase of the disease. Careful patient selection, structured patient management, and patient’s compliance is crucial for a successful WCD strategy.

## Introduction

European and American guidelines give a class I recommendation for primary preventive implantable cardioverter/defibrillator (ICD) therapy for patients with symptomatic heart failure and reduced left ventricular ejection fraction (LVEF) ≤ 35% despite optimal medical therapy [1–3]. Especially drug therapy for heart failure is usually not established at the time of first diagnosis of a cardiomyopathy, and optimization and uptitration of medical therapy is cumbersome and needs time. However, neither patients with ischemic [4, 5] nor with non-ischemic cardiomyopathy [6] benefit from early implantation of the ICD. Patients show highest benefit from an ICD if implanted 6 months after myocardial infarction [7] or even later [8]. Still, not all ICD implantations are guideline-based [9].

Nevertheless, patients have a risk for sudden cardiac death even in the early phase after diagnosis of cardiomyopathy or after myocardial infarction [10, 11].

Despite the proven mortality benefit, a relevant proportion of patients experience complications after ICD implantation [12, 13]. In the long-term course of ICD therapy, patients are particularly at risk for lead failure [14]. Over a 12-year period, 20% inappropriate shocks, 6% device infections, and 18% lead failures occur [15]. Therefore, a stressable risk stratification to identify those patients who will actually benefit from ICD therapy appears necessary. However, the only evidence-based risk marker for the decision for or against primary preventive ICD implantation remains the LVEF.

## WCD

For patients with a transient or (still) unknown risk of sudden cardiac death, a wearable defibrillator vest (WCD, LifeVest®, ZOLL) has been available for several years to prevent a sudden onset of arrhythmia. The WCD consists of a tight-fitting garment with built-in non-adhesive ECG electrodes that continuously analyze two ECG leads. When an arrhythmia is detected, an alarm cascade begins using tactile, visual, and audible alarms. If the patient is conscious during the alarm, the patient can reset the alarm by pressing two response buttons on the WCD control unit, thereby withholding any shock delivery. If, however, the patient is unconscious due to a malignant arrhythmia, the alarm cascade continues and two self-gelling shock electrodes deliver a WCD shock of up to 150 J. All recorded episodes of the WCD as well as the wearing compliance are transmitted to a remote server and can be reviewed by the attending physician (LifeVestNetwork®, ZOLL).

## Clinical Use of the WCD

WCD has been described as safe and effective [16] for detection and termination of ventricular tachyarrhythmias since the late 1990s [17]. Since then, WCD has been used in numerous clinical trials in a variety of patient populations summing up to nearly 40,000 published patients. Table 1 presents clinical trials and registries on WCD including patients with newly diagnosed cardiomyopathies only.

**Table 1:** Clinical trials and registries on WCD including newly diagnosed cardiomyopathies only

Publication	Year	Patients (*n*)	Etiology	Design	Compliance (h/day)	Cumulative wear time	Appropriate shocks	Inappropriate shocks
Feldman [60]	2004	289	Various	Prospective	n/a	Mean 3.1 months	6	6 (2%)
Saltzberg [53]	2009	258	PPCM/NICM	Retrospective	18.3/17.0	Mean 75 ± 81/56 ± 54 days	0/2	0/0
Epstein [61]	2013	8453	Post-MI	Retrospective	Median 21.8	Mean 69 ± 61 (median 57) days	146 shocks in 133 (1.6%) patients	114 in 99 patients
Zishiri [62]	2013	4958	Various	Retrospective	n/a	n/a	18 shocks in 11 patients	n/a
Duncker [56•]	2014	9	PPCM	Prospective	Mean 22.0 ± 2.4	Mean 133 ± 103 days	4	0
Kondo [63]	2015	24	Post-MI	Prospective	Median 23.1	Median 33 days	2 (8.3%)	0
Singh [41]	2015	525	NICM/ICM	Retrospective	Median 22	Median 61 days	6 patients (1.1%)	5 patients (1%)
Salehi [42]	2016	127	Alcoholic cardiomyopathy	Retrospective	Median 18.0	Median 51 days	9	18
Duncker [26•]	2017a	156	Various	Retrospective	Mean 21.7 ± 4.0	Mean 101 ± 89 days	12 shocks in 11 patients (7%)	0
Duncker [57•]	2017b	49	PPCM	Retrospective	Mean 21.4 ± 3.3	Mean 120 ± 106 days	6 patients (12%)	0
Duncker [43]	2017c	117	NICM	Retrospective	Mean 21.4 ± 4.5	Mean 101 ± 82 days	9 in 8 patients	0
Barsheshet [64]	2017	75	ICM/NICM	Retrospective	Median 18	Median 59 days	1 shock (1.3%)	0
Barraud [65]	2017	24	Post-MI	Retrospective	Median 23.5	n/a	2 in 2 patients (8.3%)	0
Erath [66]	2018	130	Various	Prospective	Mean 23	Median 42 days	2	2
Olgin [58•]	2018	2302 (1524 WCD group)	Post-MI	Randomized	Median 18.0	n/a	29 patients (WCD group)	20 patients (WCD group)

## Protected Optimization of Heart Failure Therapy

The paradox of primary prevention of sudden cardiac death is that the studies having demonstrated a survival benefit for ICD therapy included only patients in the chronic phase of heart failure with stable drug therapy [18–21]. Although the arrhythmia risk was shown to be particularly high in the early phase after myocardial infarction [10, 11], studies with ICD implantation immediately after a myocardial infarction did not show a total mortality difference between the ICD group and the control group despite significant reduction of sudden cardiac death [4, 5]. The current guidelines therefore require stable and optimal heart failure medication for at least 3 months prior to implantation of ICD in patients with heart failure and LVEF ≤ 35% [2, 3]. In everyday practice, this often results in scheduling ICD implantation 3 months after diagnosis of heart failure.

Treatment with beta-blockers and inhibition of the renin-angiotensin-aldosterone system represents the cornerstone of the medicinal heart failure therapy, which has led to a relevant reduction of morbidity and mortality [2, 22]. Nevertheless, many heart failure patients are still not optimally adjusted to the required target doses [23]. A new important drug, the angiotensin receptor-neprilysin inhibitor LCZ696, was recently established with a significant reduction in heart failure mortality [24] as well as in sudden cardiac death [25]. Drug therapy therefore seems to play a key function in heart failure treatment. The establishment of this therapy, however, requires a careful adjustment and titration of the individual classes of medication, which in turn costs quite some time in clinical routine.

The aim of the PROLONG study was to investigate the course of LVEF in patients with the new diagnosis of LVEF ≤ 35% during initiation and optimization of heart failure therapy [26•]. One hundred fifty-six patients with newly diagnosed cardiomyopathy receiving a WCD were analyzed. All patients were re-evaluated for LV function after 3 months. Patients with (1) an LVEF of 30–35%, (2) an LVEF change of ≥ 5%, or (3) not yet optimal heart failure medication were advised to prolong the WCD period. After 3 months, 88 patients still had an LVEF of ≤ 35% within the range for primary preventive ICD indication, whereas at last follow-up, this was the case for only 58 patients. Therefore, by optimizing heart failure medication, more than 30% of primary preventive ICD implantations could be avoided. Improvement of LVEF beyond 3 months in both patients after myocardial infarction and non-ischemic cardiomyopathy has also been shown in other studies [11, 27, 28].

In the PROLONG study, four patients were implanted with an ICD prematurely in external centers [26•]. All four patients showed a LVEF > 35% during follow-up, and thus no longer had a primary preventive ICD indication. However, a low arrhythmogenic risk has been described in patients recovering with LVEF [29–31]. Given the long-term risks of ICD therapy [12], it is precisely these patients who will benefit from full and optimal titration of heart failure medication before finally deciding about ICD indication. Of note PROLONG was run in the “pre-angiotensin receptor-neprilysin inhibitor” era. One may speculate that LVEF may have further improved if patients had been switched from uptitrated ACE inhibitor to angiotensin receptor-neprilysin inhibitor therapy.

At the same time, patients in the PROLONG trial show a significant risk of ventricular tachyarrhythmias both in the early stages of cardiomyopathy and in the extension phase [26•]. A total of 11 (7%) patients developed ventricular tachyarrhythmias throughout the study, so these patients benefit from temporary protection against sudden cardiac death.

The PROLONG study shows that careful optimization of heart failure therapy and prolonged waiting time can help to avoid a relevant amount of untimely ICD implantations. Nevertheless, patients still have a risk of life-threatening ventricular tachyarrhythmia during this time, and therefore, WCD should be considered. This concept can also be cost-effective in primary prevention [32].

## Ventricular Tachyarrhythmias in the Early Phase of Non-ischemic Cardiomyopathy

Even if an increased risk of ventricular tachyarrhythmias in the first few weeks after myocardial infarction is well documented [10, 11], in non-ischemic cardiomyopathy, this seems less clear.

In the long-term therapy, benefit of primary preventive ICD therapy in patients with chronic NICM has been questioned since the DANISH study [33]. In this study, 1116 patients with symptomatic heart failure and LVEF ≤ 35% under optimal heart failure medication were randomized to ICD versus no ICD. There was no significant difference in the primary endpoint of all-cause mortality between the two groups over a median follow-up of more than 5 years. Nevertheless, the rate of sudden cardiac death was significantly lower in the ICD group. However, there were subgroups that benefited from an ICD, such as younger patients (< 59 years) and lower NTproBNP level (< 1177 pg/ml). An age of ≤ 70 years was the best cutoff for highest benefit from ICD implantation [34].

Nevertheless, even with the inclusion of the DANISH trial, survival benefit was maintained in updated meta-analyses [35–38]. This in mind, studies with patients receiving a WCD reported a low incidence of ventricular tachyarrhythmias in patients with NICM [39•, 40]. Another retrospective study questioned the usefulness of WCD in non-ischemic cardiomyopathy per se, as it did not observe any WCD therapies in 254 patients with newly diagnosed NICM [41]. However, the reported incidence of 0 WCD shocks in 56.7 patient years reported in this study is not consistent with other WCD studies in patients with NICM [39•, 40, 42].

Therefore, as a subanalysis of the PROLONG study, 117 patients with newly diagnosed NICM and a LVEF ≤ 35% were investigated [43]. During a mean WCD wearing time of 101 ± 82 days, 12 ventricular tachyarrhythmias were detected in ten patients (9%). Nine appropriate WCD shocks were observed in eight patients with hemodynamically unstable ventricular tachycardia or ventricular fibrillation. Two additional patients showed more than 30 min of hemodynamically tolerated ventricular tachycardia and withheld any WCD therapies by pressing the response buttons. This event rate adds up to 38.7 tachyarrhythmia events per 100 person-years.

In contrast, in the ICD group in DANISH, providing the most actual data on VT/VF in the chronic phase of NICM, the incidence was reported to be 5.9 per 100 person-years (161 events of antitachycardia pacing or shock in cumulative 2732.5 person-years) [33]. Thereby, incidence of ventricular tachyarrhythmias was more than 6-fold higher in patients with newly diagnosed NICM and non-optimized medical therapy compared to patients in the chronic phase of NICM.

In summary, these results are not contradictory to the lower risk of long-term therapy assumed by DANISH. Patients in the PROLONG study showed newly diagnosed NICM in the phase of uptitration of drug therapy, while the DANISH study investigated patients with chronic NICM on stable medication.

## Peripartum Cardiomyopathy

Peripartum cardiomyopathy (PPCM) is a rare idiopathic cardiomyopathy leading to heart failure and left ventricular dysfunction in the last weeks of pregnancy or in the first few months after delivery [44]. Although there is often a severe deterioration of LV function at the time of diagnosis, a large proportion of patients rapidly recovers after onset of heart failure medication [45]. Significant progress has been made in recent years in terms of etiology, risk factors, and clinical management of PPCM [46, 47]. However, arrhythmia burden in patients with PPCM has been poorly studied [48], although mortality is between 2 and 15% [45, 49–52] and 38% of deaths in PPCM patients were described as sudden [50], thereby suggesting an arrhythmogenic genesis.

Using the WCD offers the possibility of continuous rhythm monitoring in the early phase of the disease as well as protection against malignant arrhythmias. An initial study of patients with PPCM using a WCD found a low risk of malignant arrhythmias in this population with no reported ventricular tachyarrhythmia in more than 35 patient years of cumulative WCD wearing time [53]. However, these results appear implausible, as other studies on PPCM, consistently reported a number of sudden cardiac deaths in the early period of the disease [50, 54, 55]. Furthermore, the design of this study is questionable because it was only retrospective data from the manufacturer database and the diagnosis of PPCM was made in patients 17 to 50 years of age who had WCD due to cardiomyopathy having been pregnant within the last 6 months [53]. This does not meet the diagnostic criteria for PPCM specified by the ESC working group [44].

In a first preliminary study, 12 patients with newly diagnosed PPCM within 1 year at the Department of Cardiology and Angiology of the Hannover Medical School were included [56•]. Patients with an LVEF ≤ 35% (*n* = 9) were recommended to wear a WCD. Two of these patients refused WCD, the remaining seven received WCD. Four episodes of ventricular fibrillation occurred in three patients during the WCD period. All four episodes immediately led to unconsciousness of the patient and were successfully detected and terminated by the WCD. This study demonstrated for the first time the potential mechanism of sudden cardiac death in this patient population of newly diagnosed PPCM, i.e., life-threatening ventricular arrhythmias. One of the main limitations this study was the small group of highly selected patients from a single tertiary center.

Therefore, based on the experience from this first monocentric study, a national multicenter study was initiated. In 16 German centers, a total of 49 patients with newly diagnosed PPCM and an LVEF ≤ 35% were identified, who were provided with a WCD after diagnosis [57•]. To date, this study represents the largest published patient population with continuous rhythm monitoring in the early stages of PPCM with reduced LVEF. During WCD period, eight ventricular arrhythmias were detected in six patients: five episodes of ventricular fibrillation, two episodes of sustained VT, and one episode of non-sustained VT. The episodes occurred between days 30 and 160 after diagnosis.

Even though the patients often have extremely poor LVEF when diagnosed with PPCM, structured and consistent heart failure therapy [47] usually leads to improvement of LVEF within a few months [45]. Nevertheless, the presented data show indicate an increased risk of ventricular tachyarrhythmias in these patients, especially during this early phase of recovery. Therefore, in patients with first diagnosis of PPCM and LVEF ≤ 35%, the WCD for a period of 3–6 months should be considered.

## VEST Study

The first prospective randomized study on WCD, the VEST trial, was recently published [58•]. two thousand three hundred and two patients with LVEF ≤ 35% after acute myocardial infarction were randomized 2:1 to patients with WCD and guideline-directed drug therapy (intervention group) and patients without WCD only receiving guideline-directed medication (control group). The primary endpoint (sudden cardiac death or death from ventricular tachyarrhythmia) was not significantly affected by WCD (1.6% vs. 2.4%, *p* = 0.18). Overall mortality as a secondary endpoint was significantly lower in the WCD group than in the control group (3.1% vs. 4.8%, *p* = 0.04). Within non-sudden deaths, significantly more stroke-related deaths occurred in the control group than in the WCD group (0.5% vs. 0%, *p* = 0.01). Other deaths were equally distributed. One major aspect has to be noticed with regard to the results: wearing compliance was remarkably low, much lower than in previously published registers, resulting in a cross-over rate of approximately 20%.

With respect to the intention-to-treat analysis, the WCD did not reduced arrhythmic death significantly. Due to the low WCD compliance, an “as treated” analysis was performed. If WCD was actually worn, arrhythmic death and mortality could be significantly reduced (arrhythmic death rate ratio, 0.43; 95% CI, 0.21 to 0.91; *p* = 0.03; mortality: rate ratio, 0.43; 95% CI, 0.21 to 0.91; uncorrected *p* = 0.03). The VEST trial shows that the WCD can reduce arrhythmic death if the device is actually worn. However, the concept of unselected providing of the WCD to every patient with a LVEF ≤ 35% after myocardial infarction without taking into account further parameters will not be supported by the results of the VEST study. The key prerequisite for the success of the WCD supply will be the wearing compliance. This finding underlines once more that further efforts need to be made in order to ensure an appropriate wearing compliance, e.g., by structured patient management programs obliging manufacturer, insurances, and attending physicians.

## Conclusions

**Fig. 1:**
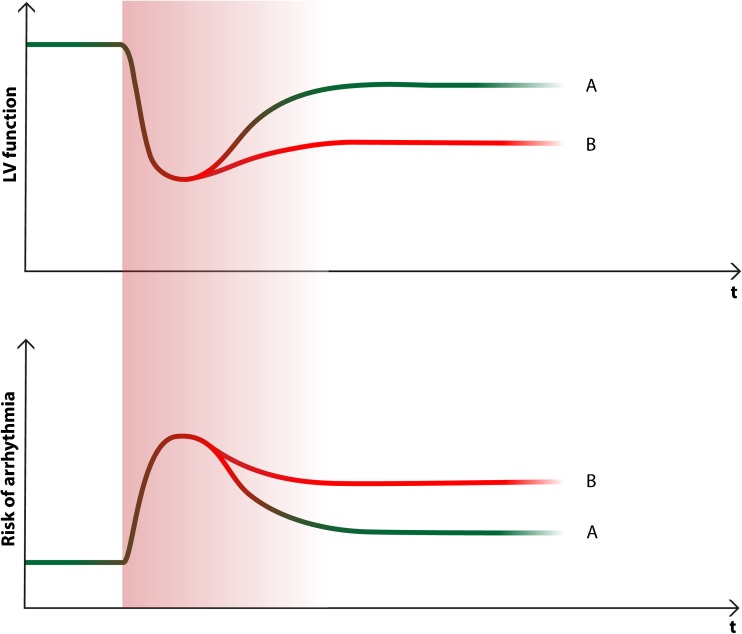
Illustration of the course of left ventricular function after diagnosis of cardiomyopathy and the associated risk of ventricular tachyarrhythmia

In the early phase of cardiomyopathy when ventricular remodeling processes take place an increased arrhythmia burden is suspected [59]. This is confirmed by several studies including patients after newly diagnosed cardiomyopathy wearing a WCD [26•, 41–43, 56, 57•, 60–66]. After transition to the chronic phase with developed and stable medication, the risk of arrhythmia may fall again, which may explain the results of the DANISH study. Figure 1 shows a conceptual illustration of the course of left ventricular function after diagnosis of cardiomyopathy and the associated risk of ventricular tachyarrhythmia. Accordingly, a WCD should be considered during the early phase after diagnosis during therapy optimization and uptitration of drug therapy.

Based on the PROLONG study, we therefore propose a standardized treatment and follow-up procedure for all patients with newly diagnosed heart failure. After diagnosis, patients receive a WCD and initiation of heart failure medication. After 3 months of individual titration of drug therapy, re-evaluation of LVEF is performed. In patients with one of the following criteria, WCD period is prolonged and re-evaluated after another 3 months: (1) LVEF between 30 and 35%, (2) delta LVEF ≥ 5%, or (3) not yet optimal dosages of heart failure medication.

Prolonging the WCD period in patients may therefore help to further optimize heart failure medication, to protect the patient from sudden cardiac death during this time, and to avoid early ICD implantation.
